# Association between the electronic frailty index and mortality in older adults: a systematic review and meta-analysis

**DOI:** 10.3389/fpubh.2026.1841492

**Published:** 2026-10-02

**Authors:** Xu Du, Yuchuan Yue, Jiang Min, Zhang Lan

**Affiliations:** The Fourth People's Hospital of Chengdu, Chengdu, China

**Keywords:** electronic frailty index, meta-analysis, mortality, older adults, systematic review

## Abstract

**Objective:**

Frailty has become an important health concern in older adults in the context of global population aging. The electronic frailty index (eFI), derived from routinely collected electronic health record data, has been increasingly used to assess frailty in clinical practice and research. However, no meta-analysis has systematically synthesized the association between eFI-defined frailty and mortality in older adults.

**Methods:**

We searched PubMed, Embase, Web of Science, Cochrane Library, ProQuest, Ovid MEDLINE Complete, CINAHL, and Scopus for studies examining the association between eFI-defined frailty and mortality in older adults, from database inception to April 1, 2025. Studies reporting hazard ratios (HRs), odds ratios (ORs), or relative risks (RRs) with 95% confidence intervals (CIs) were included. Risk of bias was assessed using the Newcastle-Ottawa Scale (NOS). Meta-analysis was performed in Stata 17.0, and heterogeneity was assessed using the I^2^ statistic.

**Results:**

Twelve studies comprising 13 cohorts were included, because one study contributed two analytical cohorts. Among these, 11 cohorts provided HR-based estimates and 2 cohort provided OR-based estimates. HR-based and OR-based estimates were analyzed separately. In the HR-based meta-analysis, eFI-defined frailty severity was associated with higher mortality risk. Compared with the non-frail group, the pooled HRs were 1.81 for mild frailty, 2.95 for moderate frailty, and 4.41for severe frailty. Substantial heterogeneity was observed across the main analyses. Subgroup analyses stratified by follow-up duration, eFI cut-off definition, study setting, and disease category suggested that the magnitude of association varied across study characteristics.

**Conclusions:**

This meta-analysis provides quantitative evidence of a graded association between eFI-defined frailty severity and mortality in older adults. These findings suggest that the eFI may be useful in frailty screening and risk stratification in geriatric care, although the substantial between-study heterogeneity warrants cautious interpretation and the results should not be interpreted as direct evidence of predictive performance.

**Systematic review registration:**

https://www.crd.york.ac.uk/PROSPERO/, identifier CRD42025632191.

## 1 Introduction

Demographic projections indicate that the proportion of adults aged 60 years and older will increase from 9.2% to 21.1% by 2050 (1). With population aging, frailty has become an increasingly significant health concern in older adults. Previous studies have shown that the prevalence of frailty varies widely, ranging from 4.0% to 59.1%, which is likely attributed to differences in study populations, care settings, and measurement instruments (2).

Frailty is generally understood as a complex geriatric syndrome characterized by age-related decline in physiological reserve and resilience, resulting in increased vulnerability to internal and external stressors (3, 4). As frailty worsens, older adults are more likely to experience adverse outcomes such as falls, hospitalization, disability, reduced quality of life, and death (5, 6). Some studies have reported that frail older adults may have a 3-to 4-fold higher risk of mortality than their non-frail counterparts (7, 8).

Several instruments have been developed to assess frailty in clinical and research settings, among which the frailty phenotype and the deficit-accumulation frailty index are the most widely used.(8, 9) The deficit-accumulation approach, originally developed within the cumulative deficit model, conceptualizes frailty as the accumulation of multiple health deficits across symptoms, diseases, disabilities, and functional impairments (10). The Rockwood frailty index and related deficit-accumulation indices can be constructed using different sources of secondary data, including population health surveys, administrative records, and electronic health records (11, 12). Although these instruments differ in data source and operational details, they are based on the same underlying principle that a greater burden of accumulated deficits reflects greater frailty severity. Survey-based evidence also supports the construct validity of this approach. For example, Casillas-Clot found that a Rockwood Frailty Index constructed from survey data was associated with subsequent decline in self-rated health among community-dwelling older adults in Southern Switzerland (13). Traditional frailty indices based on the deficit-accumulation model have also been associated with mortality in older adults, although the magnitude of these associations varies substantially across cohorts and should be interpreted in relation to study population, frailty definition, and model specification (11). This broader evidence base provides a conceptual foundation for evaluating the eFI as an electronic health record-based implementation of the cumulative deficit model, rather than as a fundamentally distinct frailty construct (12).

In routine clinical practice, however, frailty assessment based on conventional instruments often requires additional data collection, healthcare provider administration, or clinical judgment, which may limit large-scale implementation because of time burden, assessment variability, and potential subjectivity. To address these practical limitations, the electronic frailty index (eFI) was developed as an EHR-based operationalization of the cumulative deficit model using routinely collected electronic health record data (12). The eFI is therefore not a fundamentally new frailty construct, but a specific electronic implementation of the deficit-accumulation approach (10). Its distinct value lies in automated calculation, low additional data-collection burden, scalability, and the potential integration into real-world clinical information systems (14). These features provide the rationale for a dedicated quantitative synthesis focusing specifically on the association between eFI-defined frailty severity and mortality in older adults (12).

Compared with conventional frailty assessment instruments, the eFI has practical advantages for large-scale implementation because it can be generated from routinely collected electronic health record data and can incorporate multidimensional information on health deficits, functional status, cognition, comorbidities, and social factors (12, 15). Several studies have applied automated or EHR-based eFI approaches to examine associations between eFI-defined frailty and adverse health outcomes in older adults, including postoperative complications, hospitalization-related outcomes, and mortality (16–19). The eFI has also been incorporated into frailty identification and management pathways in primary care and health-system settings, reflecting its potential utility as a scalable screening and risk stratification tool (14, 20).

Nevertheless, the existing evidence remains heterogeneous. Previous systematic reviews have summarized the application of the eFI in older adults with chronic diseases, but they did not provide a category-specific quantitative synthesis of the association between eFI-defined frailty severity and mortality (21). Individual studies have also reported inconsistent findings in specific clinical contexts. For example, Hollinghurst et al. reported limited association for mild eFI-defined frailty in an emergency admission population, suggesting that the association between eFI categories and mortality may vary according to care setting, data quality, follow-up duration, and population characteristics (22). Therefore, a focused meta-analysis is needed to synthesize current evidence on whether increasing eFI-defined frailty severity is associated with progressively higher mortality risk in older adults, while also examining potential sources of heterogeneity across studies.

Accordingly, this systematic review and meta-analysis aimed to quantify the association between eFI-defined frailty severity and mortality in older adults. Specifically, we sought to: (1) estimate the pooled associations between mild, moderate, and severe eFI-defined frailty and mortality compared with non-frailty; (2) describe whether the magnitude of association increases across frailty severity categories; and (3) explore potential sources of heterogeneity according to follow-up duration, eFI cut-off definitions, study setting, and disease category.

## 2 Material and methods

This systematic review and meta-analysis was registered with PROSPERO (Registration NO: CRD42025632191) and conducted in accordance with the Preferred Reporting Items for Systematic Reviews and Meta-Analyses (PRISMA 2020) statement (23).

### 2.1 Search strategy

Comprehensive electronic searches were conducted in PubMed, Embase, Web of Science, Cochrane Library, ProQuest, Ovid MEDLINE Complete, CINAHL, and Scopus. Manual reference checks of included studies were also performed to identify additional eligible studies. The search covered the period from database inception to April 1, 2025. The search strategy combined MeSH terms and free-text keywords related to the electronic frailty index, older adults, and mortality, including “Electronic Frailty Index,” “Electronic Frailty,” “Aged,” “elder^*^,” “older^*^,” “senior^*^”, “death^*^,” “mortality,” and “mortalities”. Only English-language full-text publications were included. The full PubMed search strategy is provided in Table 1.

**Table 1:** PubMed search strategy.

Search number	Search strategy
#1	Aged [mh]
#2	elder^*^ [tiab] OR older^*^ [tiab] OR senior^*^ [tiab] OR geriatric^*^ [tiab] OR old [tiab]
#3	#1 OR #2
#4	Electronic Frailty Index [tiab] OR Frailty Tool [tiab] OR Frailty Screening *[tiab] OR Electronic Frailty Scale [tiab]
#5	Mortality [mh] OR Death [mh]
#6	Death [tiab] AND dying [tiab] OR mortality [tiab] OR death^*^[tiab]
#7	#5 OR #6
#8	#3 AND #4 AND #7

mh: MeSH; ^*^: wildcards; tiab: tittle/abstract.

### 2.2 Inclusion and exclusion criteria

Inclusion criteria were as follows: (1) studies involving older adults aged ≥65 years; (2) studies assessing frailty using the eFI or an EHR-based eFI-derived measure, with frailty categorized into non-frail/fit, mild, moderate, or severe groups where applicable; (3) observational studies reporting the association between eFI-defined frailty and mortality; (4) studies reporting effect estimates for the association between eFI-defined frailty and mortality, including hazard ratios (HRs), odds ratios (ORs), or relative risks (RRs), with corresponding 95% confidence intervals (CIs), or providing sufficient data for their extraction (5) when multiple reports used overlapping populations, the study with the largest sample size or the most complete data was included. Because HRs and ORs differ in interpretation and are commonly derived from different study designs, HR-based and OR-based estimates were synthesized and reported separately rather than combined in a single pooled analysis.

Exclusion criteria included: (1) studies with insufficient mortality data were excluded, including those that did not report HRs, ORs, RRs, corresponding 95% CIs, or sufficient information to calculate these estimates; (2) reviews, abstracts, conference papers, dissertations, editorials, commentaries, or protocols; (3) studies for which the full text was unavailable; (4) studies in which the overall association between eFI-defined frailty and mortality could not be extracted separately, including studies reporting results only within clinical-indicator-defined strata without an overall or extractable frailty-category estimate; and (5) duplicate reports or studies using overlapping populations without additional eligible data.

### 2.3 Data extraction

Two researchers independently screened the literature according to the inclusion and exclusion criteria. They first screened the titles and abstracts, and then read the full texts to determine the eligible studies. All discrepancies were resolved through discussions with a third researcher, ensuring 100% consistency in study selection and data extraction. The two researchers also double-checked and extracted information, including the first author, publication year, country, study setting, sample size, mean age, deficits, frailty level, risk estimates (HR/ OR/ RR with 95% CI), follow-up duration, and adjusted factors.

### 2.4 Risk of bias assessment

Risk of bias was assessed using the Newcastle-Ottawa Scale (NOS), which is commonly used to evaluate the methodological quality of observational studies in systematic reviews (24). The NOS evaluates three domains: selection of study groups, comparability of groups, and ascertainment of exposure or outcome. Scores range from 0 to 9, with higher scores indicating lower risk of bias. In this review, NOS scores were reported descriptively rather than classified using non-standardized quality labels.

### 2.5 Statistical analysis

Data analysis was performed using Stata 17.0. HRs and ORs with 95% CIs were used as effect measures. Because HRs and ORs differ in interpretation and underlying study design, HR-based and OR-based estimates were analyzed separately. RRs were planned to be analyzed separately if eligible studies reported them. Heterogeneity across studies was assessed using Cochran's Q test and the I^2^ statistic. A fixed-effect model was applied when *I*^2^ ≤ 50% and *P* ≥0.10; otherwise, a random-effects model was used. Leave-one-out sensitivity analysis was performed to assess the influence of individual studies on pooled estimates. Subgroup analyses were conducted to explore potential sources of heterogeneity, including follow-up duration, eFI cut-off definitions, study setting, and disease category. Publication bias or small-study effects were assessed using funnel plots, Egger's test, and the trim-and-fill method where appropriate. Statistical significance was set at *P* < 0.05.

Because only three pooled frailty-category estimates were available for mild, moderate, and severe frailty, a formal dose-response meta-analysis was not performed. Instead, the gradient in mortality risk across eFI-defined frailty severity categories was described qualitatively. Given that most included studies adjusted only for age and sex, the pooled estimates were interpreted as associations rather than fully adjusted independent effects, and residual confounding by comorbidities, socioeconomic status, baseline disease burden, and healthcare access was considered a key limitation of the evidence base.

## 3 Results

### 3.1 Literature search

Electronic database searches identified 2,138 records, and 10 additional records were identified through manual reference searching, yielding 2,148 records in total. After removal of 654 duplicate records, 1,494 records were screened by title and abstract. Of these, 1,374 records were excluded because they were not relevant to the review question. A total of 120 full-text articles were assessed for eligibility, of which 108 were excluded for the following reasons: conference abstracts or reviews (*n* = 50), ineligible study populations (*n* = 3), mortality not reported as an outcome (*n* = 32), full text unavailable (*n* = 13), and insufficient mortality data (*n* = 10). Finally, 12 studies comprising 13 cohorts were included in the systematic review and meta-analysis. The study selection process is shown in Figure 1.

**Figure 1:**
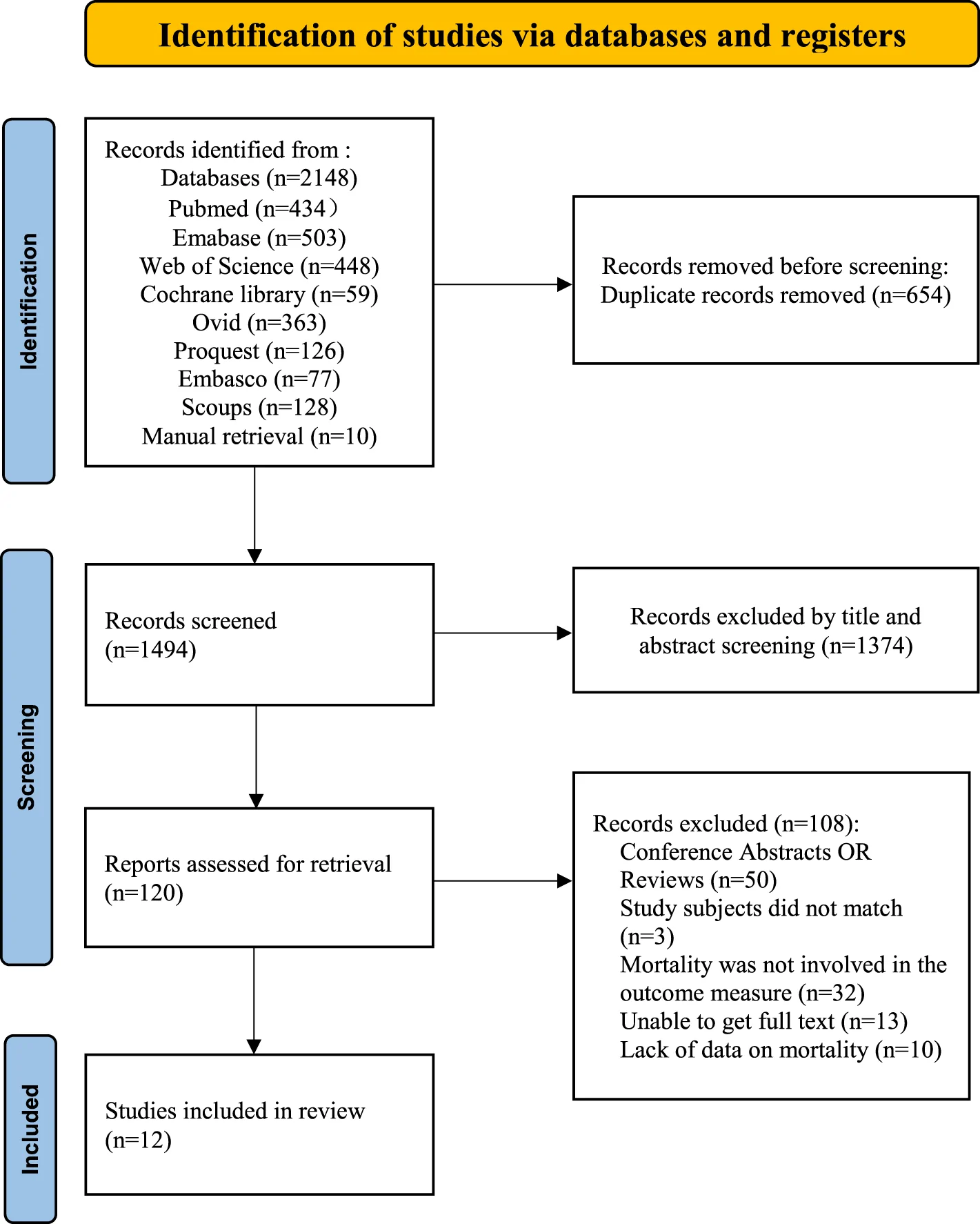
PRISMA flow diagram of study selection.

### 3.2 Characteristics of included studies

The baseline characteristics of the included studies are presented in Table 2. A total of 12 studies comprising 13 cohorts were included, because one study contributed two validation cohorts. The publication years ranged from 2016 to 2025, and sample sizes ranged from 3,980 to 536,955 participants. The proportion of female participants varied substantially across studies, ranging from 1.4% to 60.2%, suggesting marked differences in sex composition and indicating that sex distribution may have contributed to between-study heterogeneity. Seven studies were conducted in primary care settings and five in hospital settings. Among the 13 cohorts, 11 cohorts reported HRs and 2 cohorts reported ORs for the association between eFI-defined frailty and mortality; HR-based and OR-based estimates were analyzed separately. The number of eFI deficits ranged from 31 to 48: one study used 31 deficits, eight studies used 36 deficits, one study used 45 deficits, and two studies used 48 deficits. All included studies categorized frailty severity into fit or non-frail, mild, moderate, and severe groups. However, the cut-off values used to define frailty categories varied across studies: eight studies used 0–0.12, 0.12–0.24, 0.24–0.36, and ≥0.36; two studies used 0–0.15, 0.15–0.20, 0.20–0.25, and ≥0.25; one study used 0–0.20, 0.20–0.30, 0.30–0.40, and ≥0.40; and one study used 0.125–0.159, 0.159–0.204, and >0.205. Follow-up duration for mortality outcomes ranged from in-hospital mortality to 5 years.

**Table 2:** Baseline characteristics of the included studies.

References	Country	Setting	Sample size	Mean age	Female (%)	Defects	Frailty level	HR/RR/OR (95%CI)	Mortality prediction follow-up period	Adjusted factors
Andrew Clegg et al. (12)^a^	UK	Primary care	516,007	75.0 ± 7.3	56%	36	Mild (0.12–0.24)	HR = 1.86 (1.78–1.95) HR = 1.73 (1.68–1.77) HR = 1.66 (1.63–1.69)	1 year 3 years 5 years	Age and sex
							Moderate (0.24–0.36)	HR = 3.02 (2.88–3.16) HR = 2.70 (2.63–2.77) HR = 2.54 (2.49–2.60)		
							Severe (>0.36)	HR = 4.50 (4.26–4.76) HR = 4.06 (3.93–4.19) HR = 3.84 (3.74–3.94)		
Andrew Clegg et al. (12)^b^	UK	Primary care	207,720	75.0 ± 7.3	55%	36	Mild (0.12–0.24)	HR = 1.92 (1.81–2.04) HR = 1.77 (1.71–1.83) HR = 1.72 (1.68–1.77)	1 year 3 years 5 years	Age and sex
							Moderate (0.24–0.36)	HR = 3.10 (2.91–3.31) HR = 2.78 (2.68–2.89) HR = 2.64 (2.57–2.72)		
							Severe (>0.36)	HR = 4.52 (4.16–4.91) HR = 3.99 (3.79–4.20) HR = 3.83 (3.68–3.99)		
Clark DuMontier et al. (25)	USA	Primary care	4,924	≥65	1.4%	31	Mild (0.20–0.30)	HR = 1.54 (1.27–1.86)	2.4 years	Age, sociodemographic factors and disease risk measures
							Moderate (0.30–0.40)	HR = 1.95 (1.61–2.37)		
							Severe (>0.40)	HR = 2.50 (2.05–3.04)		
Fabienne Hershkowitz Sikron et al. (30)	Israeli	Primary care	120,986	73.9 ± 7.0	54.3%	36	Mild (0.12–0.24)	HR = 2.07 (1.93–2.23)	1 year	Sex, age and socio–economic status
							Moderate (0.24–0.36)	HR = 3.35 (3.07–3.66)		
							Severe (>0.36)	HR = 4.40 (3.96–4.89)		
Francesc Orfila et al. (28)	Spanish	Primary care	253,684	76.3 ± 7.9	59.5%	36	Mild (0.12–0.24)	HR = 1.69 (1.61–1.78)	2 years	Age and sex
							Moderate (0.24–0.36)	HR = 3.42 (3.25–3.61)		
							Severe (>0.36)	HR = 7.09 (6.72–7.49)		
Joe Hollinghurst et al. (26)	UK	Primary care	469,000	74.9 ± 7.4	55%	36	Mild (0.12–0.24)	HR = 2.04 (1.96–2.13) HR = 1.83 (1.79–1.87) HR = 1.80 (1.77–1.83)	1 year 3 years 5 years	Age and sex
							Moderate (0.24–0.36)	HR = 3.41 (3.27–3.57) HR = 2.98 (2.91–3.06) HR = 2.85 (2.80–2.91)		
							Severe (>0.36)	HR = 5.32 (5.03–5.62) HR = 4.59 (4.44–4.75) HR = 4.31 (4.19–4.42)		
Joe Hollinghurst et al. (22)	UK	Primary care	126,600	79.3 ± 8.4	53.1%	36	Mild (0.12–0.24)	HR = 1.00 (0.96–1.04)	3 months	Age and sex
							Moderate (0.24–0.36)	HR = 1.08 (1.03–1.12)		
							Severe (>0.36)	HR = 1.16 (1.10–1.21)		
Jonathan K L Mak et al. (27)	Switzerland	Hospital	3,980	81.6 ± 8.5	60.2%	48	Mild (0.15–0.20)	HR = 1.56 (1.17–2.08) HR = 1.48 (1.18–1.85)	1 month 6 months	Age and sex
							Moderate (0.20–0.25)	HR = 3.02 (2.30–3.07) HR = 2.69 (2.17–3.34)		
							Severe (>0.25)	HR = 4.70 (3.54–6.23) HR = 4.20 (3.35–5.26)		
Jonathan K L Mak et al. (31)	Switzerland	Hospital	13,188	83.1 ± 8.4	53.8%	48	Mild (0.15–0.20)	HR = 2.63 (1.93–3.60) HR = 1.94 (1.66–2.26)	1 month 6 months	Age and sex
							Moderate (0.20–0.25)	HR = 4.83 (3.57–6.54) HR = 3.45 (2.97–4.01)		
							Severe (>0.25)	HR = 9.87 (7.31, 13.3) HR = 5.78 (4.96, 6.74)		
							Moderate (0.24–0.36)	HR = 2.11 (1.73–2.58) HR = 2.57 (2.19–3.02)		
							Severe (>0.36)	HR = 2.87 (2.24–3.66) HR = 3.48 (2.85–4.25)		
Chris Wilkinson et al. (29)	UK	Hospital	536,955	73.8	54.2%	36	Mild (0.12–0.24)	HR = 1.52 (1.27–1.82)	2 years	Age, sex, smoking status and multiple deprivation IMD and practice identifier IDs
							Moderate (0.24–0.36)	HR = 2.47 (2.06–2.94)		
							Severe (>0.36)	HR = 4.09 (3.43–4.89)		
Stow et al. (43)	UK	Primary care	9,204	≥75	55.3%	36	Mild (0.12–0.24)	OR = 1.80 (1.60–2.02)	3 months	Age and sex
							Moderate (0.24–0.36)	OR = 3.19 (2.83–3.59)		
							Severe (>0.36)	OR = 4.72 (4.16–5.36)		
Dana Shiffer et al. (32)	Italy	Hospital	21,537	77.44 ± 6.97	49.3%	45	Mild (0.125–0.159)	OR = 2.61 (1.19–6.16)	In–hospital mortality	Age and sex
							Moderate (0.159–0.204)	OR = 5.41 (2.77–11.79)		
							Severe (>0.205)	OR = 14.15 (7.92–28.11)		

a External validation queue.

b Internal validation queue.

### 3.3 Risk of bias results

NOS scores ranged from 6 to 8 across the included studies: one study scored 8, nine studies scored 7, and two studies scored 6 (Table 3). Most studies had clearly defined eFI exposure categories, adequate representativeness of the study population, and ascertainment of mortality outcomes. However, the comparability domain varied across studies. Eleven studies adjusted for age, whereas only one study adjusted for additional covariates beyond age and sex. This limited adjustment indicates potential residual confounding and should be considered when interpreting the pooled associations.

**Table 3:** Risk of bias assessment of the included studies using the Newcastle-Ottawa Scale (NOS).

References	Selection				Comparability		Outcome/Exposure			Total score
	Representativeness of the exposed cohort/is the case definition adequate?	Selection of the non–exposed cohort/representativeness of the cases	Ascertainment of exposure/selection of controls	Demonstration that outcome of interest was not present at start of study/definition of controls	Comparability of cohorts on the basis of the design or analysis		Assessment of outcome/ascertainment of exposure	Was follow–up long enough for outcomes to occur/same method of ascertainment for cases and controls	Adequacy of follow up of cohorts/non–response rate	
					a) Study controls for age	b) Study controls for any additional factor				
Clegg et al. (12)	1	0	1	1	1	0	1	1	1	7
Dana Shiffer et al. (32)	1	0	1	1	1	0	1	1	0	6
Clark DuMontier et al. (25)	1	0	1	1	1	0	1	1	1	7
Joe Hollinghurst et al. (26)	1	0	1	1	1	0	1	1	1	7
Joe Hollinghurst et al. (22)	1	0	1	1	1	0	1	1	0	7
Matthew T H Lowry et al. (18)	1	0	1	1	1	0	1	1	1	7
Jonathan K L Mak et al. (31)	1	0	1	1	1	0	1	1	1	7
Jonathan K L Mak et al. (27)	1	0	1	1	1	0	1	1	1	7
Francesc Orfila et al. (28)	1	0	1	1	1	1	1	1	1	8
Fabienne Hershkowitz Sikron et al. (30)	1	0	1	1	1	0	1	1	1	7
Stow et al. (43)	1	1	1	1	0	1	0	1	0	6
Chris Wilkinson et al. (29)	1	0	1	1	1	0	1	1	1	7

### 3.4 Meta-analysis of studies using HR

#### 3.4.1 Meta-analysis for mild frailty

Ten studies comprising 11 HR-based cohorts reported the association between mild eFI-defined frailty and mortality (12, 18, 22, 25–31). Because substantial heterogeneity was observed across cohorts, a random-effects model was applied. Compared with non-frail older adults, mild eFI-defined frailty was associated with a higher risk of mortality (pooled HR = 1.81, 95% CI: 1.70–1.93, *P* < 0.001; *I*^2^ = 83.9%) (Figure 2).

**Figure 2:**
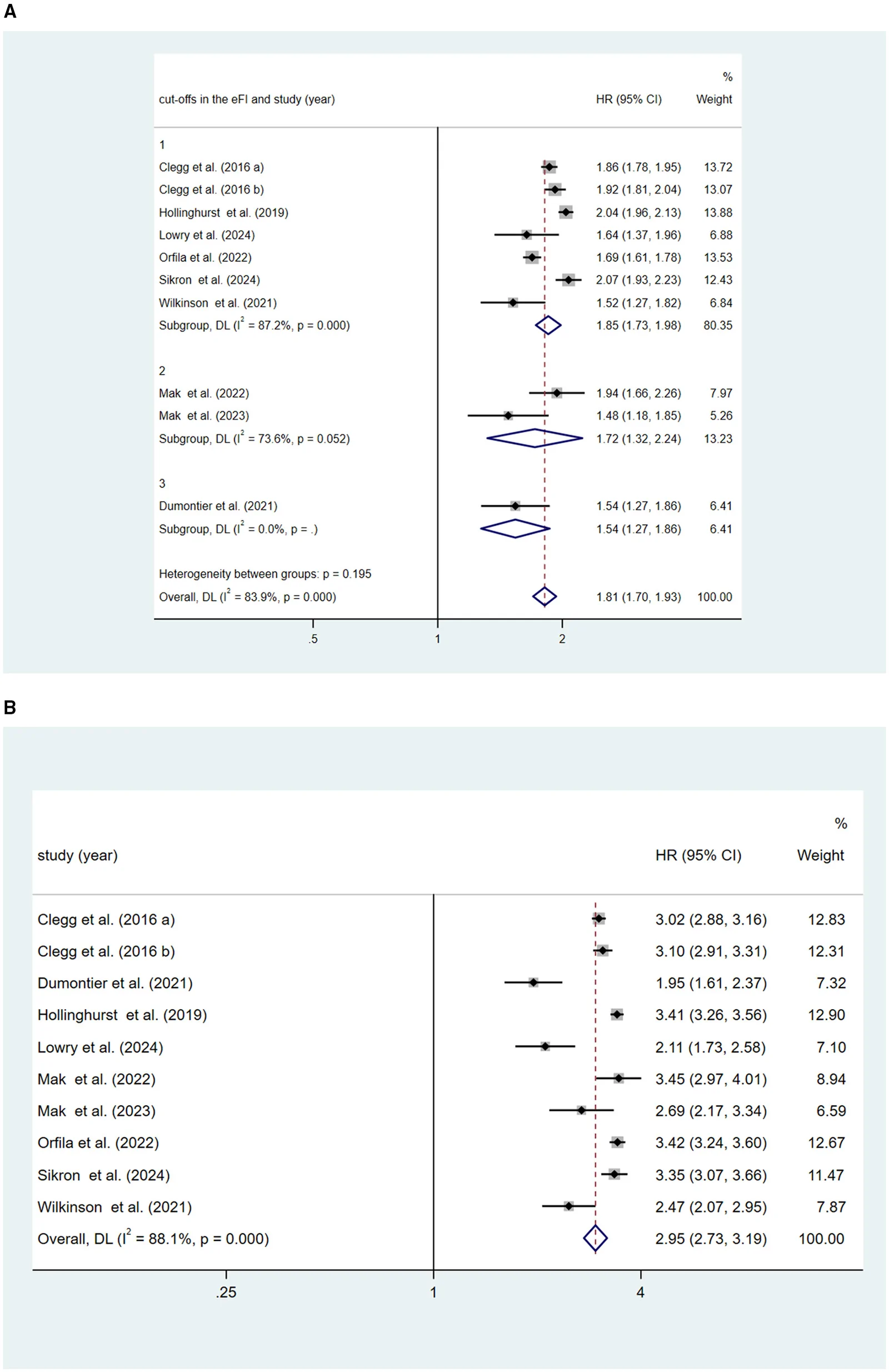
Forest plot for mild eFI-defined frailty and mortality among HR-based studies. Forest plot of the relationship between moderate frailty as assessed by eFI and mortality. HR, hazard ratio; 95% CI, 95% confidence interval; **(a)** external validation cohort; **(b)** internal validation cohort.

#### 3.4.2 Meta-analysis for moderate frailty

Ten studies comprising 11 HR-based cohorts reported the association between moderate eFI-defined frailty and mortality. Because substantial heterogeneity was observed across cohorts, a random-effects model was applied. Compared with non-frail older adults, moderate eFI-defined frailty was associated with a higher risk of mortality (pooled HR = 2.95, 95% CI: 2.73–3.19, *P* < 0.001; *I*^2^ = 88.1%) (Figure 3).

**Figure 3:**
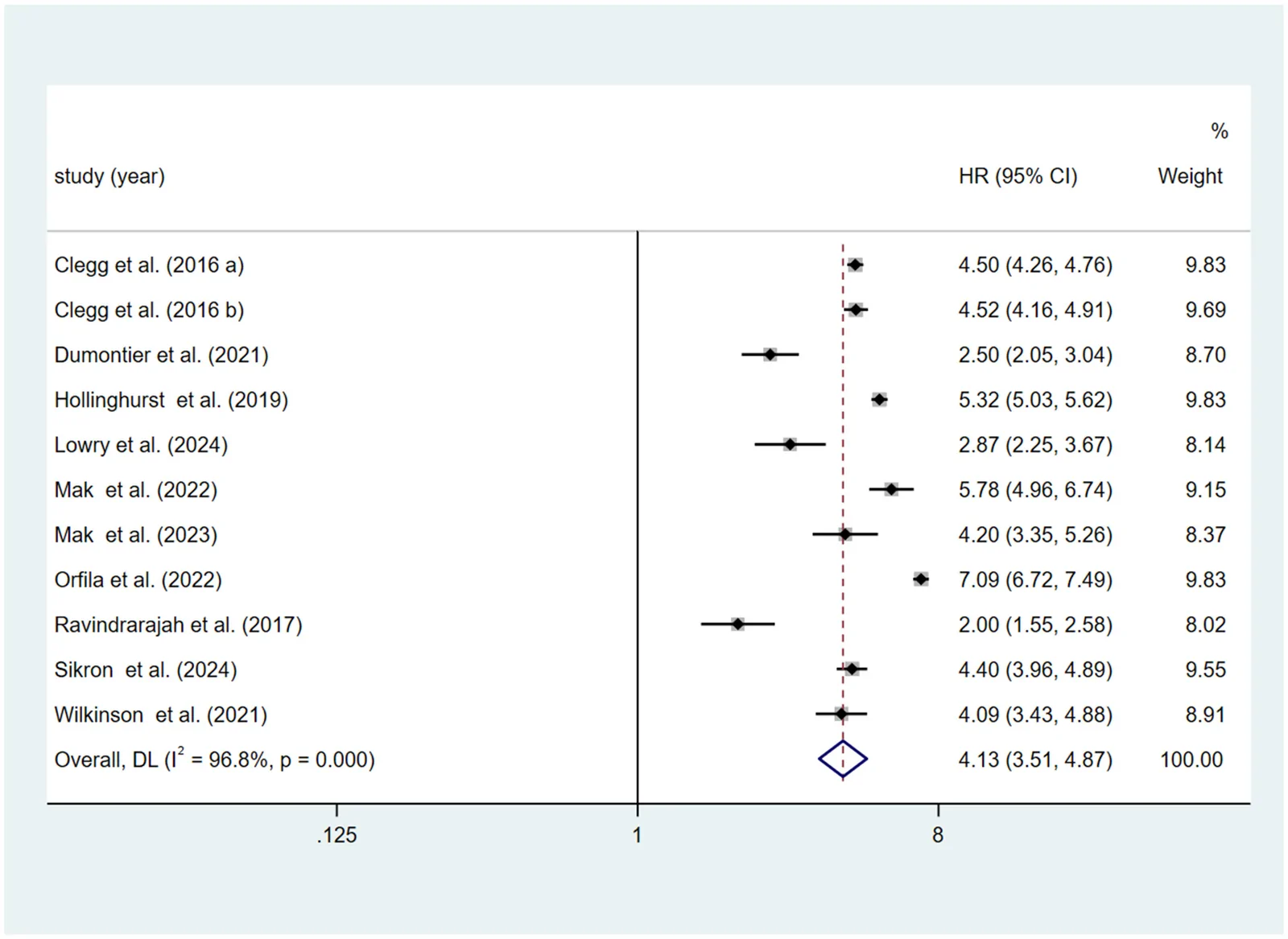
Forest plot for moderate eFI-defined frailty and mortality among HR-based studies.

#### 3.4.3 Meta-analysis for severe frailty

Ten studies comprising 11 HR-based cohorts reported the association between severe eFI-defined frailty and mortality. Because substantial heterogeneity was observed across cohorts, a random-effects model was applied. Compared with non-frail older adults, severe eFI-defined frailty was associated with a higher risk of mortality (pooled HR = 4.41, 95% CI: 3.77–5.17, *P* < 0.001; *I*^2^ = 96.5%) (Figure 4).

**Figure 4:**
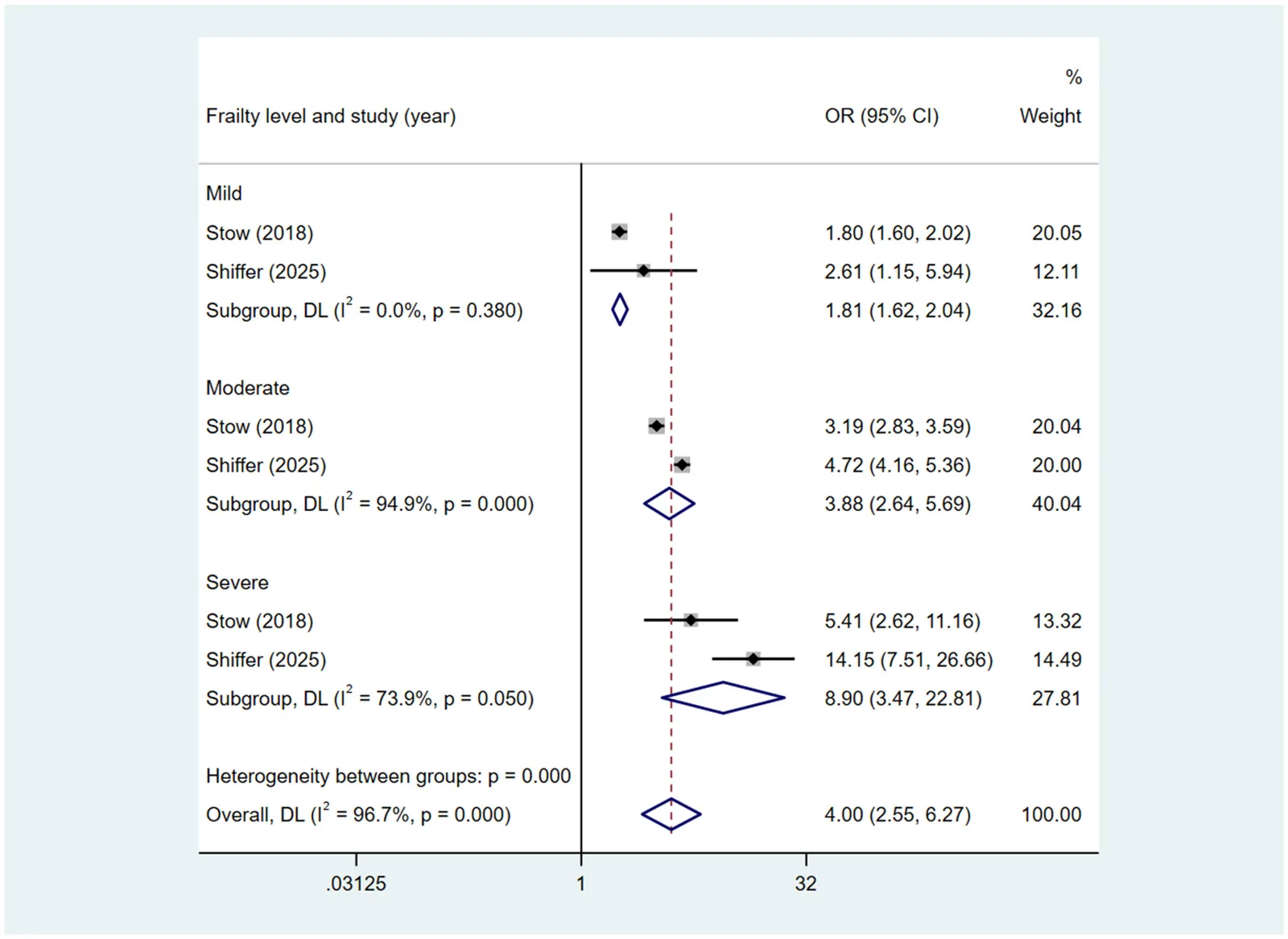
Forest plot for severe eFI-defined frailty and mortality among HR-based studies.

### 3.5 Meta-analysis of studies using OR

Two studies reported ORs for the association between eFI-defined frailty and mortality (31, 32). Compared with non-frail older adults, higher mortality was observed in the mild (OR = 1.81, 95% CI: 1.62–2.04), moderate (OR = 3.88, 95% CI: 2.64–5.69), and severe (OR = 8.90, 95% CI: 3.47–22.81) frailty groups (Figure 5). However, because the OR-based analysis included only two studies, and the estimate for severe frailty had a wide confidence interval, these findings may be interpreted as exploratory and supplementary evidence rather than with the same level of confidence as the HR-based pooled estimates.

**Figure 5:**
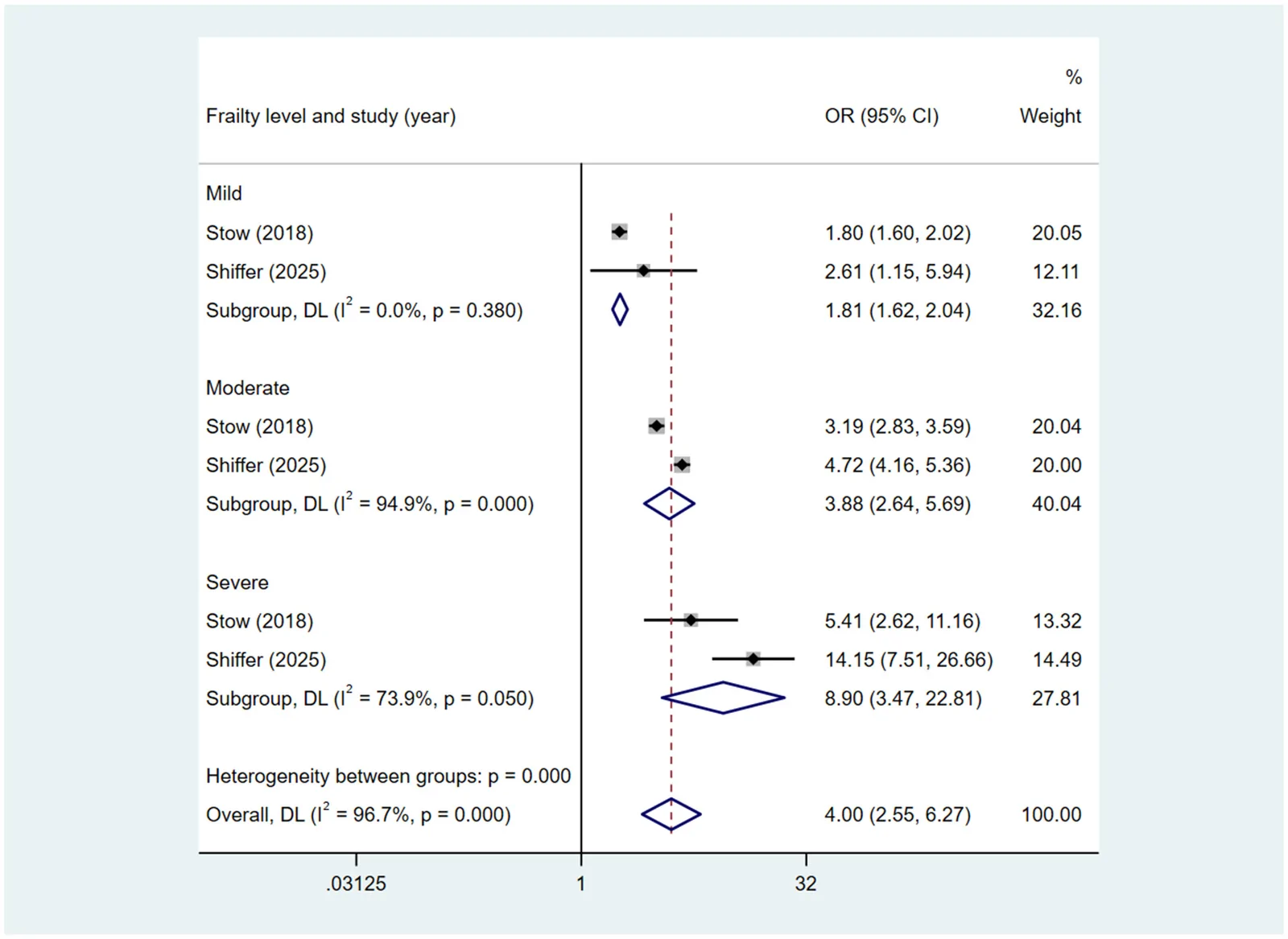
OR-based analysis of eFI-defined frailty and mortality.

### 3.6 Descriptive gradient across frailty severity categories

Across the HR-based analyses, the magnitude of association increased progressively from mild to moderate and severe eFI-defined frailty. Compared with non-frail older adults, the pooled HRs were 1.81 for mild frailty, 2.95 for moderate frailty, and 4.41 for severe frailty, suggesting a graded association between eFI-defined frailty severity and mortality. Because this pattern was based on three pooled category-specific estimates, it was interpreted descriptively rather than as evidence from a formal dose-response regression model.

### 3.7 Subgroup analysis of studies reporting HR

To further explore potential sources of heterogeneity among HR-based studies, subgroup analyses were conducted according to follow-up duration, eFI cut-off values, study setting, and disease category. The complete subgroup-specific estimates are summarized in Appendix 1 of the Supplementary Material.

#### 3.7.1 Subgroup analysis for mild frailty

For mild eFI-defined frailty, subgroup analyses were conducted according to follow-up duration, eFI cut-off values, study setting, and disease category. The complete subgroup-specific estimates are summarized in Appendix 1 of the Supplementary Material. The highest pooled estimates were observed in studies with ≤ 1 year of follow-up (HR = 1.94, 95% CI: 1.80–2.10, *P* < 0.001), studies using the standard eFI cut-off scheme (HR = 1.85, 95% CI: 1.73–1.98, P < 0.001), primary care settings (HR = 1.87, 95% CI: 1.75–2.01, *P* < 0.001), and all-system or mixed disease populations (HR = 1.91, 95% CI: 1.79–2.04, *P* < 0.001). Overall, mild eFI-defined frailty was associated with higher mortality risk across most subgroup categories, although the magnitude of association varied across subgroups.

#### 3.7.2 Subgroup analysis for moderate frailty

For moderate eFI-defined frailty, subgroup analyses were conducted according to follow-up duration, eFI cut-off values, study setting, and disease category. The complete subgroup-specific estimates are summarized in Appendix 1 of the Supplementary Material. The highest pooled estimates were observed in studies with ≤ 1 year of follow-up (HR = 3.18, 95% CI: 2.75–3.68, *P* < 0.001), studies using the alternative eFI cut-off scheme (HR = 3.08, 95% CI: 2.42–3.93, *P* < 0.001), primary care settings (HR = 3.10, 95% CI: 2.87–3.36, *P* < 0.001), and all-system or mixed disease populations (HR = 3.27, 95% CI: 3.10–3.45, *P* < 0.001). Overall, moderate eFI-defined frailty was associated with higher mortality risk across most subgroup categories, although the magnitude of association varied across subgroups.

#### 3.7.3 Subgroup analysis for severe frailty

For severe eFI-defined frailty, subgroup analyses were conducted according to follow-up duration, eFI cut-off values, study setting, and disease category. The complete subgroup-specific estimates are summarized in Appendix 1 of the Supplementary Material. The highest pooled estimates were observed in studies with ≤ 1 year of follow-up (HR = 4.56, 95% CI: 4.11–5.05, *P* < 0.001), studies using the 0.15–0.20, 0.20–0.25, and ≥0.25 eFI cut-off scheme (HR = 4.98, 95% CI: 3.65–6.81, *P* < 0.001), primary care settings (HR = 4.57, 95% CI: 3.75–5.58, *P* < 0.001), and all-system or mixed disease populations (HR = 5.19, 95% CI: 4.37–6.16, *P* < 0.001). Overall, severe eFI-defined frailty was associated with higher mortality risk across most subgroup categories, although the magnitude of association varied across subgroups.

### 3.8 Sensitivity analysis

Sensitivity analysis was performed using the leave-one-out method to assess the influence of individual studies on the pooled HR estimates. Hollinghurst et al. (23) had a noticeable influence on the magnitude of the pooled estimates and between-study heterogeneity (22). This may be related to its specific emergency admission population, retrospective administrative data source, and differences in the eFI assessment window. After excluding this study in sensitivity analysis, the direction of association remained consistent across mild, moderate, and severe frailty categories. Therefore, Hollinghurst et al was retained in the primary and subgroup meta-analyses, and its influence was considered when interpreting heterogeneity.

### 3.9 Publication bias

Funnel plot asymmetry and Egger's test (*P* < 0.001) suggested small-study effects or potential publication bias across the mild, moderate, and severe frailty groups. However, given the substantial between-study heterogeneity (*I*^2^ > 84%) observed in the main analyses, this asymmetry may reflect heterogeneity-driven small-study effects rather than publication bias alone. The trim-and-fill method did not identify additional missing studies, and the pooled estimates remained unchanged after this procedure. Nevertheless, this finding may be interpreted cautiously because trim-and-fill methods may be unreliable in the presence of substantial heterogeneity. Therefore, small-study effects or potential publication bias cannot be excluded.

## 4 Discussion

This meta-analysis synthesized evidence from 12 studies and found that increasing severity of eFI-defined frailty was associated with progressively higher mortality risk in older adults. Compared with non-frail older adults, mild, moderate, and severe eFI-defined frailty were associated with higher mortality risk, with pooled HRs of 1.81, 2.95, and 4.41, respectively. This pattern suggests a graded association between eFI-defined frailty severity and mortality. However, substantial heterogeneity was observed across the main analyses, with I^2^ values ranging from 83.9% to 96.5%. Therefore, the precise numerical values of the pooled HRs may be interpreted cautiously. These estimates are more appropriately understood as summary associations across clinically and methodologically diverse studies, rather than as stable or universally generalizable clinical effect sizes.

It is important to distinguish association from clinical prediction. The included studies mainly reported HRs or ORs, which quantify associations between eFI-defined frailty and mortality. These measures do not directly evaluate whether the eFI can accurately discriminate between individuals who will and will not die. No discrimination or calibration metrics, such as AUC, C-index, sensitivity, specificity, or calibration plots, were synthesized in this review. Therefore, the present findings indicate that eFI-defined frailty severity is associated with mortality risk, but they do not establish individual-level clinical predictive accuracy.

The present findings may be interpreted within the broader cumulative deficit framework. Traditional frailty indices and the eFI are both based on the deficit-accumulation model and therefore measure closely related frailty constructs (16, 21, 32). Compared with traditional frailty indices or survey-based Rockwood-type indices, the distinct value of the eFI does not lie in representing a fundamentally different construct, but in its use of routinely collected electronic health record data, automated calculation, scalability, and potential for real-world clinical implementation (14). Evidence from deficit-accumulation indices derived from different data sources, including survey-based indices, supports the construct stability of this framework across operationalizations (9, 11, 13). Thus, this study does not demonstrate the superiority of the eFI over other frailty instruments; rather, it provides a quantitative synthesis of the association between eFI-defined frailty categories and mortality in settings where EHR data are available.

The graded association observed in this meta-analysis is clinically plausible. Frailty reflects multisystem vulnerability, reduced physiological reserve, chronic inflammation, sarcopenia, cognitive decline, multimorbidity, and reduced tolerance to clinical stressors (33–38). As accumulated deficits increase, older adults may become less resilient to acute illness and chronic disease burden, which may explain the progressively higher mortality risk observed across mild, moderate, and severe eFI-defined frailty categories. However, because the included studies differed in population characteristics, follow-up duration, eFI cut-off values, study setting, and covariate adjustment, these biological and clinical interpretations may remain cautious.

Subgroup analyses suggested that the magnitude of association varied across follow-up duration, eFI cut-off definitions, healthcare settings, and disease categories. Shorter follow-up studies tended to show larger pooled estimates, which may reflect the short-term vulnerability of frail older adults to acute clinical deterioration. The proportion of female participants also varied substantially across included studies, ranging from 1.4% to 60.2%, suggesting that differences in sex composition may have contributed to between-study heterogeneity. These subgroup findings may not be interpreted as definitive evidence of effect modification, because several strata included a limited number of studies and residual heterogeneity remained (26).

The finding that primary care settings showed higher pooled estimates than hospital settings also requires cautious interpretation. One possible explanation is that primary care cohorts include large community-dwelling populations with substantial multimorbidity, variable healthcare access, and longer observation windows, making frailty-related mortality differences more apparent (39). However, an alternative explanation is that hospital-based studies may involve shorter follow-up periods, more intensive monitoring, and more timely clinical intervention, which could attenuate observable mortality differences between frailty categories. Therefore, setting-based differences may not be interpreted as evidence that the eFI performs better in one care setting than another without further comparative validation.

From a clinical perspective, the graded association observed in this meta-analysis may support the use of the eFI as a scalable tool for frailty screening and mortality risk stratification in health systems with routinely collected electronic health record data (12, 14). However, specific tiered intervention strategies may be interpreted as clinical extrapolations based on existing frailty management guidance, such as British Geriatrics Society recommendations, rather than as intervention effects directly demonstrated by this meta-analysis (20, 40). The present study provides evidence that eFI-defined frailty severity is associated with mortality risk, but it does not determine which interventions may be applied to each frailty category or whether such interventions reduce mortality.

Potential misclassification of eFI-defined frailty may also be considered. Preliminary evidence from a small single-center study suggests possible misclassification in EHR-based frailty identification, but this finding requires confirmation in larger multicenter validation studies (41). Incomplete records, coding errors, delayed data updates, and variability in deficit definitions may affect eFI classification and contribute to heterogeneity across studies (41, 42).

Several limitations may be considered. First, inconsistency in eFI cut-off definitions across included studies is a major limitation. Although many studies used the standard thresholds, others used substantially different cut-off values. Therefore, the categories of mild, moderate, and severe frailty may not represent equivalent populations across studies. This is a conceptual comparability problem, not merely a statistical source of heterogeneity, and it directly limits the interpretability of category-specific pooled estimates. Second, substantial between-study het13gerogeneity was present across the main analyses, limiting the interpretation of pooled HRs as single summary effects. Third, residual confounding is a major concern because most studies adjusted only for age and sex, whereas adjustment for comorbidities, socioeconomic status, baseline disease burden, healthcare access, and other clinical factors was limited and inconsistent. As a result, the pooled HRs may overestimate the independent association between eFI-defined frailty and mortality. Fourth, the OR-based analysis included only two studies, and the estimate for severe frailty had a wide confidence interval; therefore, these findings may be regarded as supplementary evidence. Fifth, the review was restricted to English-language publications, which may have led to omission of relevant evidence from non-English-speaking countries or regions, introducing language bias and affecting the completeness and reproducibility of the review. Finally, funnel plot asymmetry and significant Egger's tests suggest possible small-study effects or publication bias, although these patterns may also reflect heterogeneity-driven asymmetry.

Future research may use standardized eFI cut-off definitions, report more comprehensive covariate-adjusted models, and include formal prediction performance metrics if the clinical predictive utility of the eFI is to be evaluated. Future studies may also explore dynamic data sources, wearable devices, and machine learning approaches to refine frailty assessment, but these may be regarded as speculative future directions rather than conclusions derived from the present meta-analysis.

## 5 Conclusions and implications

This meta-analysis provides quantitative support for a graded association between eFI-defined frailty severity and mortality in older adults. Mild, moderate, and severe eFI-defined frailty were associated with progressively higher mortality risk compared with non-frailty. However, substantial between-study heterogeneity, inconsistent eFI cut-off definitions, possible small-study effects or publication bias, and limited confounder adjustment warrant cautious interpretation. Because this review did not synthesize discrimination or calibration metrics, these findings may not be interpreted as evidence that the eFI has independent individual-level predictive accuracy for mortality. In clinical settings requiring short-term mortality risk assessment, older adults with eFI-defined frailty may require closer monitoring and risk management, but specific intervention strategies may be guided by existing clinical guidelines and validated in future studies (20, 40).

## Data Availability

The original contributions presented in the study are included in the article, further inquiries can be directed to the corresponding author.
