# Supplementary material for: Association between the electronic frailty index and mortality in older adults: a systematic review and meta-analysis
Source: Front Public Health. 2026 Oct 2;14:1841492. doi: 10.3389/fpubh.2026.1841492 (PMC13672487; doi:10.3389/fpubh.2026.1841492)
Supplement: Supplementary file 1 [file Supplementary_file_1.docx]

**Appendix 1. Supplementary subgroup analysis figures**

These figures correspond to the subgroup analyses described in Section 3.7 of the manuscript.

**Fig. 1a.** Subgroup analysis by follow-up duration.

**Fig. 1b.** Subgroup analysis by eFI cut-off definition.

**Fig. 1c.** Subgroup analysis by study setting.

**Fig. 1d.** Subgroup analysis by disease category.

**Fig. 1.** Subgroup analyses for mild eFI-defined frailty.

**Fig. 2a.** Subgroup analysis by follow-up duration.

**Fig. 2b.** Subgroup analysis by eFI cut-off definition.

**Fig. 2c.** Subgroup analysis by study setting.

**Fig. 2d.** Subgroup analysis by disease category.

**Fig. 2.** Subgroup analyses for moderate eFI-defined frailty.

**Fig. 3a.** Subgroup analysis by follow-up duration.

**Fig. 3b.**Subgroup analysis by eFI cut-off definition.

**Fig. 3c.** Subgroup analysis by study setting.

**Fig. 3d.** Subgroup analysis by disease category.

**Fig. 3.** Subgroup analyses for severe eFI-defined frailty.

Abbreviations: eFI, electronic frailty index; HR, hazard ratio; CI, confidence interval.
